# Strain-Controlled Quantum Dot Fine Structure for Entangled Photon Generation at 1550 nm

**DOI:** 10.1021/acs.nanolett.1c04024

**Published:** 2021-12-13

**Authors:** Thomas Lettner, Samuel Gyger, Katharina D. Zeuner, Lucas Schweickert, Stephan Steinhauer, Carl Reuterskiöld Hedlund, Sandra Stroj, Armando Rastelli, Mattias Hammar, Rinaldo Trotta, Klaus D. Jöns, Val Zwiller

**Affiliations:** † Department of Applied Physics, KTH Royal Institute of Technology, Albanova University Centre, Roslagstullsbacken 21, 106 91 Stockholm, Sweden; ‡ Department of Electrical Engineering, KTH Royal Institute of Technology, Electrum 229, 164 40 Kista, Sweden; § Research Center for Microtechnology, Vorarlberg University of Applied Sciences, Campus V, Hochschulstrasse 1, 6850 Dornbirn, Austria; ⊥ Institute of Semiconductor and Solid State Physics, Johannes Kepler University, 4040 Linz, Austria; ∥ Department of Physics, Sapienza University of Rome, Piazzale A. Moro 5, 00185 Rome, Italy

**Keywords:** semiconductor quantum dots, entangled photons, strain tuning, fine-structure splitting, quantum state tomography, telecom wavelengths, single-photon source

## Abstract

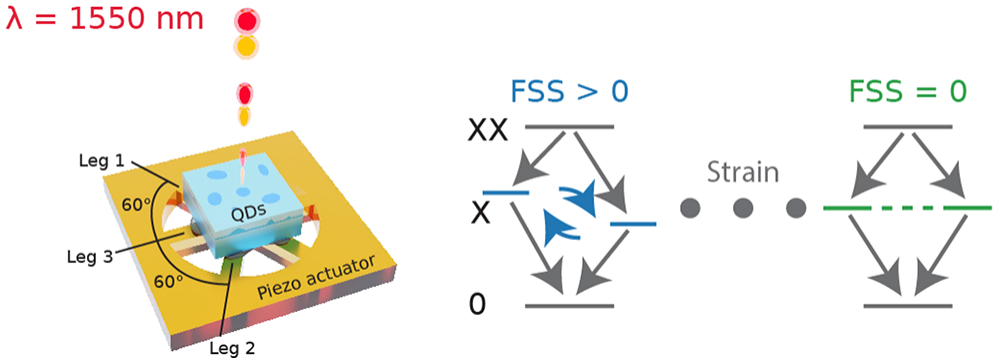

Entangled photon generation at 1550 nm in the telecom C-band is of critical importance as it enables the realization of quantum communication protocols over long distance using deployed telecommunication infrastructure. InAs epitaxial quantum dots have recently enabled on-demand generation of entangled photons in this wavelength range. However, time-dependent state evolution, caused by the fine-structure splitting, currently limits the fidelity to a specific entangled state. Here, we show fine-structure suppression for InAs quantum dots using micromachined piezoelectric actuators and demonstrate generation of highly entangled photons at 1550 nm. At the lowest fine-structure setting, we obtain a maximum fidelity of 90.0 ± 2.7% (concurrence of 87.5 ± 3.1%). The concurrence remains high also for moderate (weak) temporal filtering, with values close to 80% (50%), corresponding to 30% (80%) of collected photons, respectively. The presented fine-structure control opens the way for exploiting entangled photons from quantum dots in fiber-based quantum communication protocols.

Entangled photon sources are crucial building blocks for the realization of flying qubits in the emerging quantum internet.^1^ The generation of single and entangled photons in the telecom C-band (1530–1565 nm) is of great scientific and technological importance: operation in this wavelength range allows for compatibility with existing telecom infrastructure and long-range transmission due to the low losses in deployed optical fibers. Among the envisioned applications are entanglement-based quantum key distribution,^2,3^ clock synchronization,^4^ quantum computer networks,^5^ and cloud quantum computing.^6^ Furthermore, nonclassical states of light in the near-infrared are an important resource for low-energy communication, lidar,^7^ and super-resolution microscopy.^8,9^ Semiconductor quantum dots (QDs) are outstanding nonclassical light sources in terms of single-photon purity^10−13^ and generation of highly entangled photon pairs.^12−18^ On-demand entangled photon generation with a concurrence of 91.4% and a fidelity of 95.2% has recently been demonstrated^19^ with InAs QDs on a metamorphic buffer layer emitting in the telecom C-band.^20^

Semiconductor QDs can emit polarization-entangled photon pairs through the decay cascade^21^ from the biexciton (XX) state to the ground state via the intermediate exciton (X) level, leading to the Bell state . However, the presence of anisotropy lifts the X level degeneracy and the entangled state changes into . The time-varying phase is directly proportional to the fine-structure splitting (FSS) of the X level via ϕ(*t*) = *t* FSS/*ℏ*, with *t* being the time elapsed between the XX and X decay, and results in the X state oscillating between two eigenstates after the emission of the XX photon. Still, a high degree of entanglement has been measured,^17^ but this requires elaborate temporal postselection using expensive detectors with sufficiently high time resolution.

The FSS is unique to every single QD and depends on its shape and strain environment. The development of a well-controlled low-strain QD growth has allowed the fabrication of highly symmetric GaAs QDs^22^ with very low initial FSS of only a few μeV. Similar efforts have been made in the telecom C-band for InAs QDs grown on InP^23^ and on a metamorphic buffer layer.^24^ Postgrowth tuning via external fields has been successfully applied to control the FSS of semiconductor QDs.^25,26^ In particular, piezoelectric actuators have been shown to provide full control of the in-plane strain tensor in a reversible way.^27^ With a patterned triaxial strain actuator^28,29^ that allows tuning the FSS to zero, a nearly dephasing-free source of on-demand entangled photon pairs with a wavelength around 780 nm has been realized.^17^

In this work, we erase the FSS of a single InAs QD emitting in the telecom C-band and study the impact on the entangled state temporal evolution. To achieve this, we integrate our QD sample on a micromachined six-legged piezoelectric actuator enabling full control of the QD anisotropy via strain. Then, we perform time-resolved quantum state tomography with a system time resolution of 55 and 64 ps for the two measurement channels. This allows us to investigate the entangled state fidelity and concurrence for different time bins and study the oscillation of the state as we approach zero FSS. By lowering the FSS, we observe an increase in the oscillation period by an order of magnitude. In the low FSS regime, we measure a high concurrence of 87.5 ± 3.1% (fidelity of 90.0 ± 2.7%), which remains close to 80% even for moderate temporal binning of 512 ps.

The device is sketched in Figure 1a. The sample consists of InAs QDs on a metamorphic buffer InGaAs layer grown on GaAs(001) by metal–organic vapor-phase epitaxy^30^ and is mechanically thinned to 40 μm thickness. We use polymer-based bonding to integrate the sample onto a micromachined piezoelectric actuator with three pairs of laser-cut legs individually contacted with gold electrodes arranged at 60° with respect to each other.^29^ This configuration allows one to tune the magnitude and the strain anisotropy in the sample and with this the splitting of the X state. The piezoelectric material is Pb(Mg_1/3_Nb_2/3_)O_3_–PbTiO_3_ (PMN–PT), known to provide high strain values at cryogenic temperatures. The inset in Figure 1b shows an optical microscope image of the fabricated device. The sample is mounted on a three-axis piezo-based positioner stack (attocube) in a closed-cycle cryostat (Montana cryostation) and cooled below 20 K. We use a confocal μ-photoluminescence (μ-PL) setup and excite the sample into the QD p-shell with a pulsed picosecond laser at 1470 nm. The emission is collected using an objective (attocube LT-APO/NIR NA = 0.8) and coupled to an optical fiber (SMF-28). Figure 1b shows the μ-PL spectrum of the studied QD acquired with a pixel-to-pixel resolution of 25 μeV (Acton SP2750i, 830 lines/mm grating, Princeton Instruments OMA V InGaAs array detector), with the transitions of X, XX, and trion (T) indicated.

**Figure 1:**
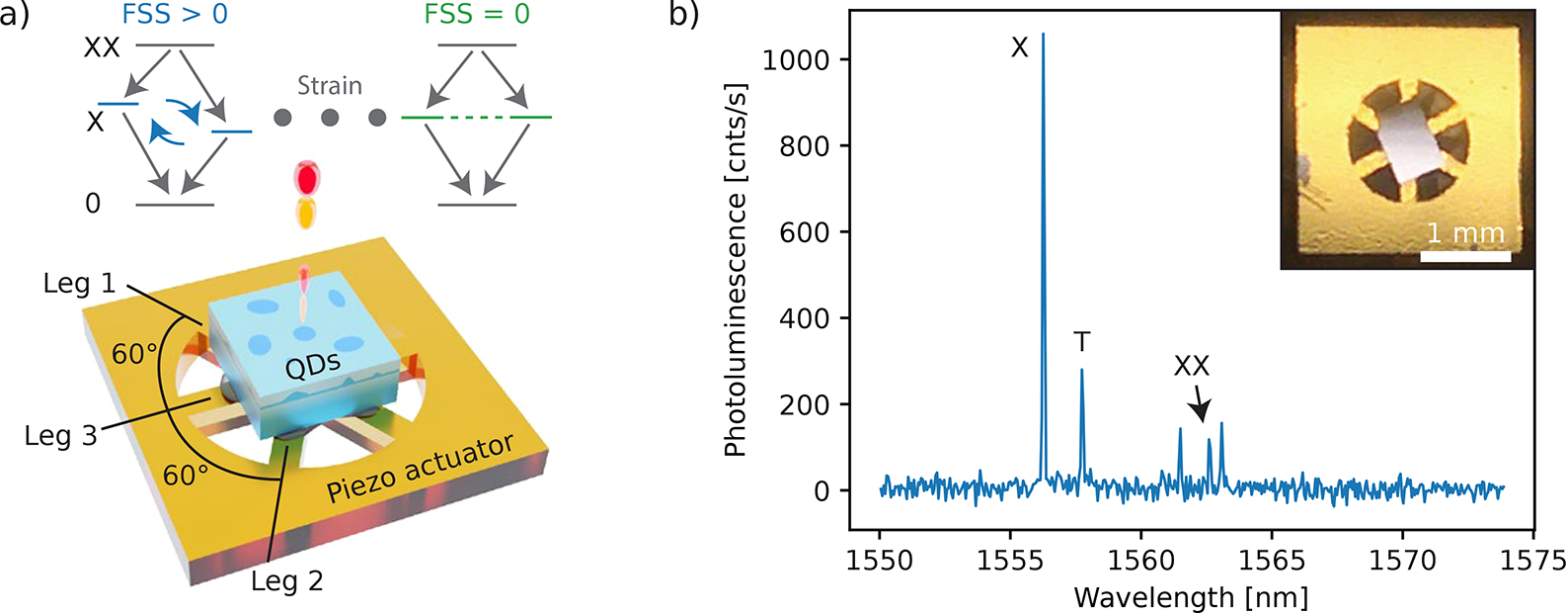
Device concept and realization: (a) Schematic of the six-legged piezoelectric actuator fully controlling the in-plane strain in a thinned sample containing of InGaAs QDs. Applying the right combination of voltages allows tuning of the FSS of the X transition to zero. (b) μ-PL of the studied QD with the relevant transitions labeled. Inset: Photograph of the fabricated device with the micromachined piezoelectric actuator (gold) and the QD sample (gray rectangle) in the center.

Then, we perform polarization-resolved μ-PL measurements for varying voltages applied to the individual pairs of legs and record the peak positions from a Gaussian fit as a function of polarization angle (Figure 2a). A fit of these data with a sine function allows us to extract the FSS and polarization angle of the high energy component of the X emission from the magnitude and phase of the oscillation, respectively. We observe that by only using leg 2 and keeping leg 1 at 0 V, we tune the FSS through a minimum of around 5 μeV; see the blue symbols in the top panel of Figure 2b. This is an indication of the coherent coupling between the two bright X states.^31^ To suppress this coupling and bring the FSS to zero, we set leg 1 to 470 V and repeat again the voltage sweep on leg 2, as shown with orange symbols in Figure 2. Then, at around 400 V applied to leg 2, the FSS approaches the resolution of our experimental apparatus. This can be understood considering that the voltage applied to leg 1 (470 V) rotates the principal anisotropy axes of the QD along/perpendicular to the principal axes of the stress induced by leg 2. As a result, leg 2 only tunes the FSS magnitude without modifying the polarization direction of the X emission doublet. For this reason, we observe a sharp 90° rotation of the polarization direction of the high-energy component of the X emission, practically indicating a crossing of the two lines at the point where the FSS is minimal^25^ (Figure 2b). More information about the tuning characterization and fitting procedure can be found in the Supporting Information.

**Figure 2:**
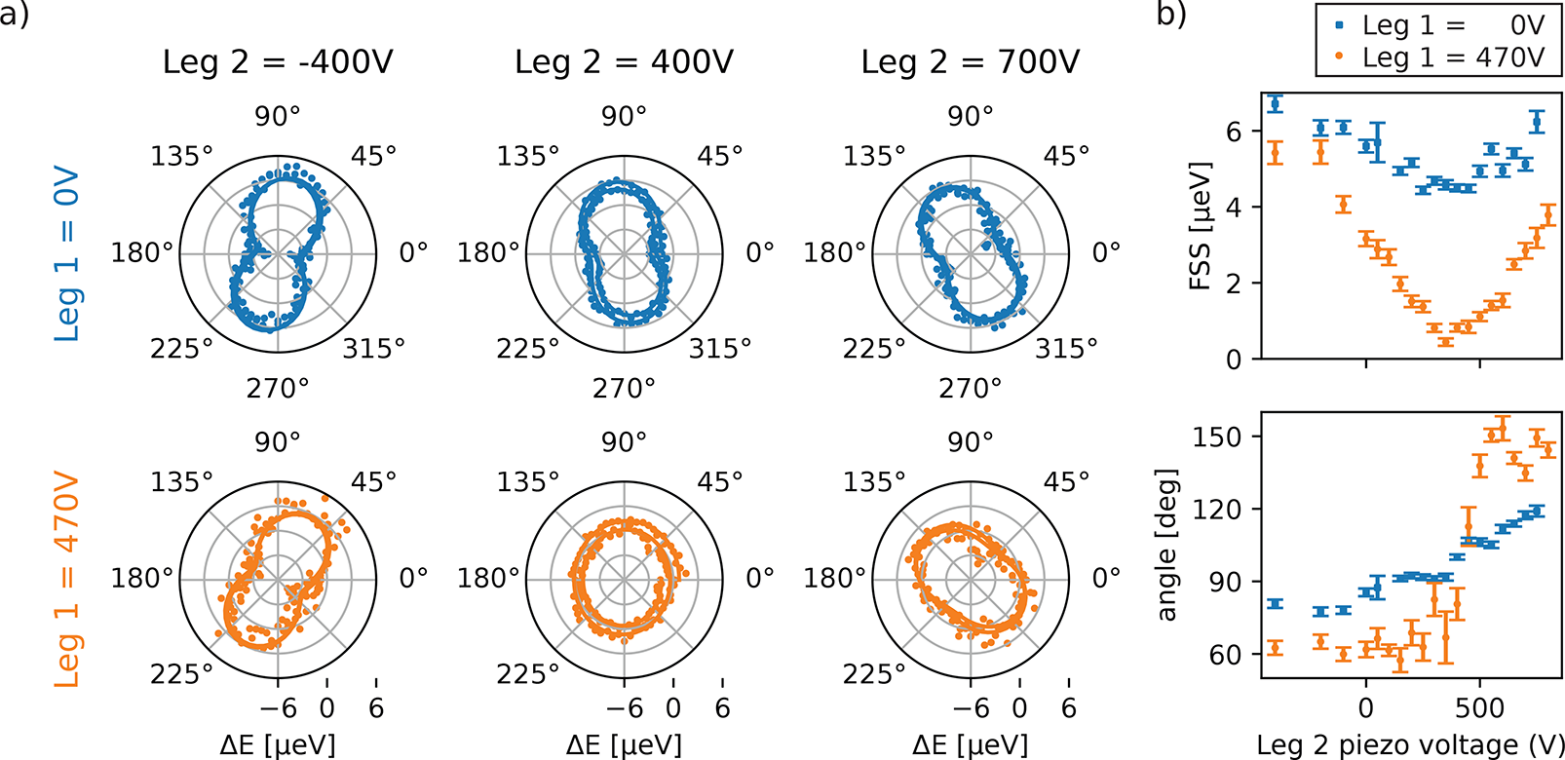
Strain control of the FSS by ramping voltages applied across the actuator legs. (a) Polar plots show the X energy deviation Δ*E* obtained from polarization resolved μ-PL measurements as a function of polarization angle. (b) Leg 2 tuning curves, while leg 1 is kept at 0 V (blue squares) and set to 470 V (orange circles) such that the quantum dot anisotropy is aligned to the application direction of leg 2. The FSS (angle) is derived from the amplitude (phase) of a sine fit to Δ*E*.

Next, we select X and XX transitions using a home-built transmission spectrometer with a bandwidth of 13 GHz and two polarization-controlled outputs each coupled to a channel of a superconducting nanowire single-photon detector (SNSPD) system (Single Quantum EOS, 30 and 15 ps detector timing jitter for the respective channels). We time-tag (PicoQuant HydraHarp 400) and analyze the signals (ETA^32^) and obtain polarization-dependent cross-correlation histograms. We perform these measurements for all 36 two-photon polarization measurement bases over a period of 3 h and then perform a density matrix reconstruction for each individual time bin using the maximum likelihood method.^33^ Then we rotate the resulting matrices using a general retarder transformation in order to compensate for birefringence in the collection path of the setup using the same procedure as in ref (24). Next, we choose three different sets of piezoelectric actuator voltages for high, medium, and low FSS corresponding to 4.8 ± 0.4, 3.7 ± 0.1, and 0.4 ± 0.1 μeV, respectively, according to measurements of polarization resolved μ-PL. We evaluate the fidelity to the state Φ^+^ as a function of time delay between X and XX emission and observe oscillations due to the time evolution of the X state populated by the decay of the XX state. The oscillation period is longest for the lowest FSS setting (Figure 3a). A sine fit to each set of data allows one to extract an oscillation period and corresponding FSS value. The high FSS setting results in fast oscillations with a 321.6 ± 1.3 ps period corresponding to 12.9 ± 0.1 μeV. With medium FSS, the period increases to 632.8 ± 1.7 ps (6.5 ± 0.1 μeV). The low FSS case almost erases these oscillations as the period reaches 3.9 ± 0.5 ns (1.1 ± 0.1 μeV), which is well above the lifetime of the X transition of ≈2 ns. In this low-anisotropy regime, we reach a maximum fidelity of 90.0 ± 2.7% (concurrence of 87.5 ± 3.1%) at time delay *t* = 96 ps using a temporal bin width of 32 ps. The density matrix for the 32 ps time window corresponding to the maximum fidelity is presented in Figure 3b,c and features almost exclusively entries in the off-diagonals and a vanishing imaginary part, as expected for a Φ^+^ state. The density matrices for the medium and high FSS settings show similar characteristics; see Figures S3 and S4 in the Supporting Information.

**Figure 3:**
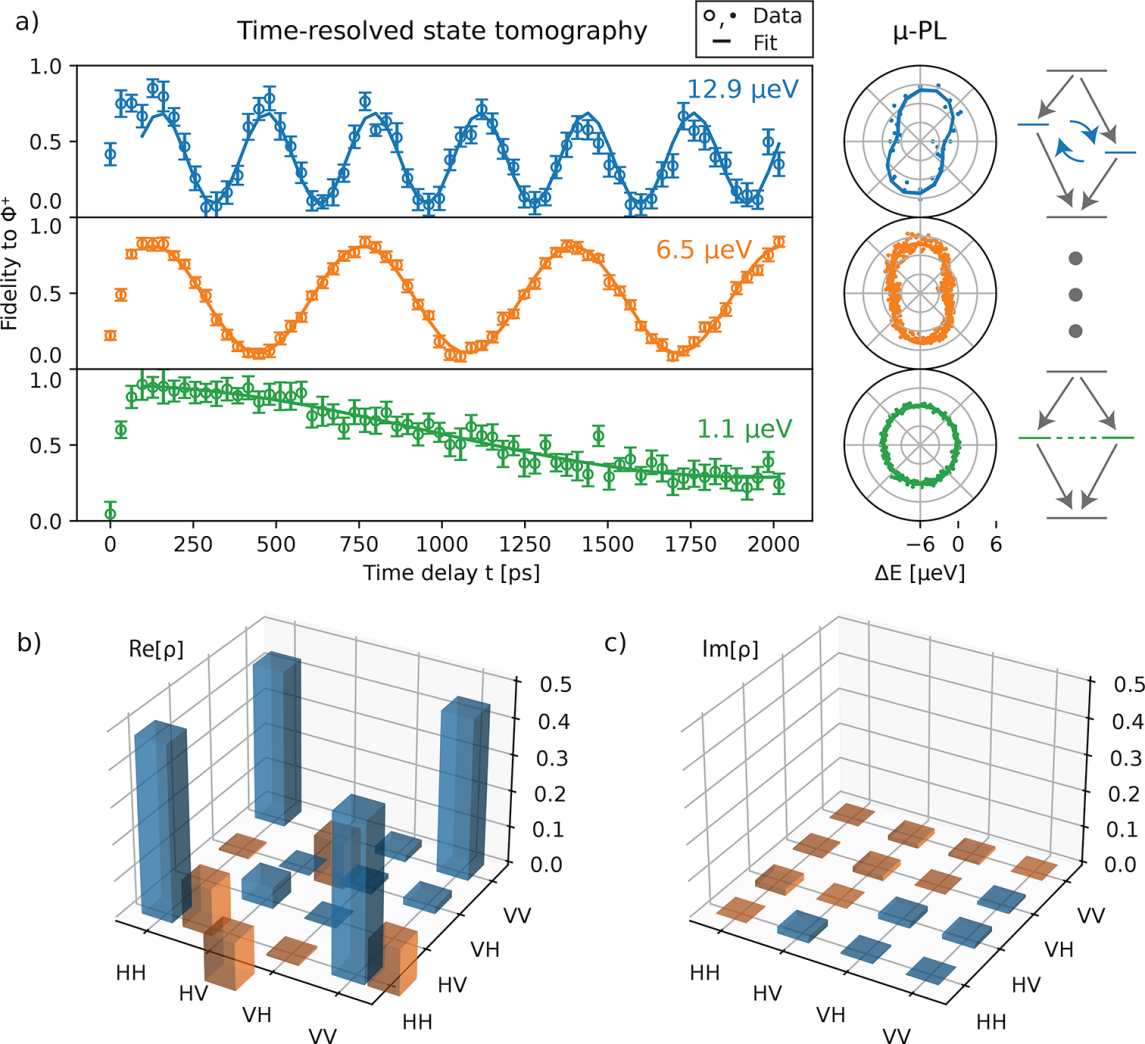
Time-resolved characterization of the entangled state Φ^+^ for varying FSS. (a) Dependence of the fidelity on the time delay between emission from X with respect to XX for three different FSS settings. Polar plots show the corresponding polarization-resolved PL measurement results. (b,c) Real and imaginary parts of the density matrix for the lowest FSS setting with peak fidelity of 90.0 ± 2.7% at 96 ps.

The QD emits entangled photon pairs in all three cases, which we can confirm through the high time resolution of our setup. Applying such strong temporal postselection, however, excludes a large fraction of the accumulated correlations, effectively reducing the efficiency of the entangled photon source. To counteract, we would have to reduce the amount of time filtering applied to the data by increasing the time bin width. However, this has a negative impact on the (time-averaged) entanglement concurrence unless the FSS is sufficiently low.

This can be seen in Figure 4, where we evaluate the concurrence as a function of additional time binning applied to the cross-correlation histograms. We continuously increase the binning width used for the data analysis with ETA in multiples of 2, starting from the initial 32 ps. Then we record the concurrence from the time bin with the highest concurrence for the three previously used FSS settings. For both the high and medium FSS settings, an increasing bin size results in a quick drop in concurrence from initially close to 80% to less than 10% for a 2.048 ns bin width. The situation is different for the low FSS setting, where the concurrence stays close to the initial 80%, even for moderate binning up to 512 ps. For 2.048 ns, we still obtain a maximum concurrence of 50%. The moderate bin width of 512 ps already corresponds to 30% of the accumulated correlations, and for 2.048 ns, we can utilize nearly all counts (80%). From this, the importance of reaching low FSS becomes evident as it allows one to increase the capability of the QD to emit predominantly the desired Bell state, in this case Φ^+^.

**Figure 4:**
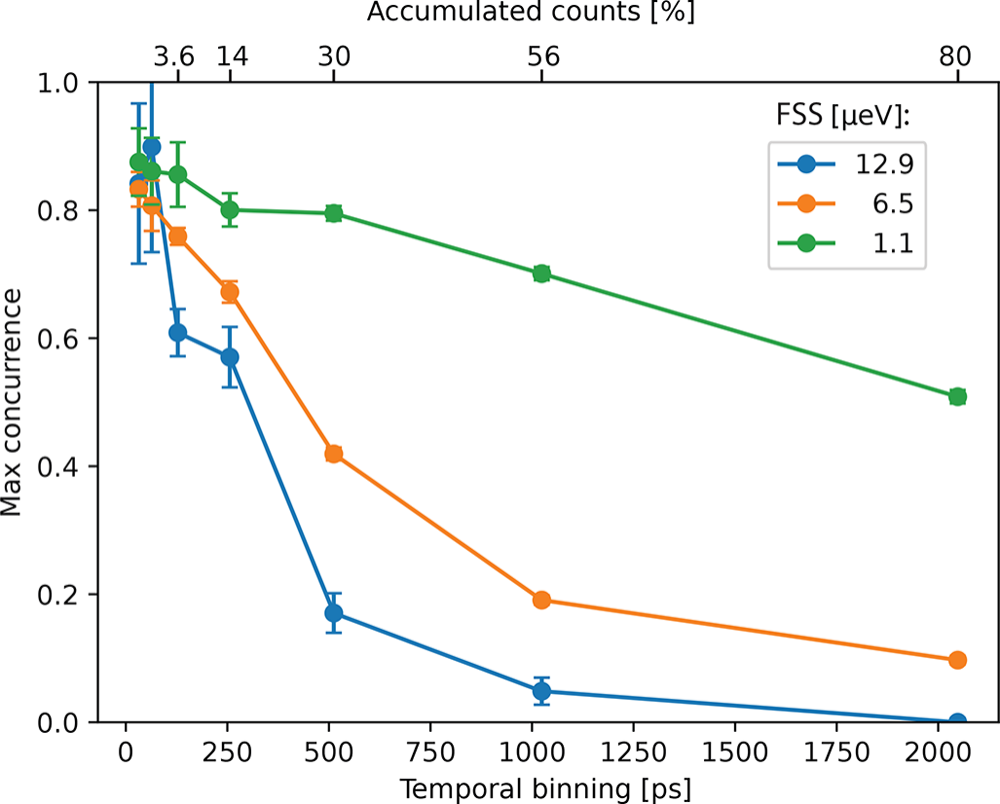
Impact of binning used for temporal post selection on the concurrence for varying FSS. Points represent the maximum concurrence evaluated from all the time bins, with the bin width increasing in multiples of 2. Lines connecting points are guides to the eye. Top scale: Correlation counts accumulated within the corresponding time bin width, relative to the total amount of detected correlations from the XX–X cascade.

We have demonstrated reversible control of the fine-structure splitting of a QD and generation of highly entangled photons in the telecom C-band. This has been facilitated by combining the quantum emitter with a six-legged piezoelectric actuator device. The strain-tuning capabilities of the device enabled us to manipulate and reduce the FSS down to 0.4 ± 0.1 μeV. We observed a marked increase in the oscillation period of the entangled state to ≈4 ns corresponding to a residual FSS of 1.1 ± 0.1 μeV. We attribute the difference to the fine structure obtained from the PL to birefringence of the optical elements in the setup collection path which could be compensated for using a suitable phase retarder.^34^ In the low FSS regime, we measure entangled photon emission with 90.0 ± 2.7% fidelity and 87.5 ± 3.1% concurrence. We expect these values to improve further by (i) eliminating the residual FSS and (ii) shortening of the X transition lifetime by using a cavity.^35^ As a consequence of the low FSS, we obtain a concurrence of 80% for moderate binning of 512 ps, which corresponds to 30% of the accumulated correlations. Our findings also demonstrate that the time evolution of the entangled state hinders observing near-unity entanglement concurrence unless time resolution is sufficiently high. The insights we have obtained are crucial for further enhancing semiconductor QD properties and employing them as high-performance entangled photon sources in the telecom C-band.

## Supporting Information Available

Strain-controlled quantum dot fine-structure for entangled-photon generation at 1550 nm: sample growth and device fabrication; piezoelectric actuator tuning characterization, second-order correlation of exciton transition, and density matrices for medium and large fine structure (PDF)

## References

[ref1] Kimble H. J. The Quantum Internet. Nature 2008, 453, 1023–1030. 10.1038/nature07127. 18563153

[ref2] Ekert A. K. Quantum Cryptography Based on Bell’s Theorem. Phys. Rev. Lett. 1991, 67, 661–663. 10.1103/PhysRevLett.67.661. 10044956

[ref3] Gisin N.; Ribordy G.; Tittel W.; Zbinden H. Quantum Cryptography. Rev. Mod. Phys. 2002, 74, 145–195. 10.1103/RevModPhys.74.145.

[ref4] Valencia A.; Scarcelli G.; Shih Y. Distant clock synchronization using entangled photon pairs. Appl. Phys. Lett. 2004, 85, 2655–2657. 10.1063/1.1797561.

[ref5] Wehner S.; Elkouss D.; Hanson R. Quantum internet: A vision for the road ahead. Science 2018, 362, eaam9288 10.1126/science.aam9288. 30337383

[ref6] Devitt S. J. Performing Quantum Computing Experiments in the Cloud. Phys. Rev. A: At., Mol., Opt. Phys. 2016, 94, 032329 10.1103/PhysRevA.94.032329.

[ref7] Zhuang Q.; Zhang Z.; Shapiro J. H. Entanglement-Enhanced Lidars for Simultaneous Range and Velocity Measurements. Phys. Rev. A: At., Mol., Opt. Phys. 2017, 96, 040304 10.1103/PhysRevA.96.040304.

[ref8] Nagata T.; Okamoto R.; O’Brien J. L.; Sasaki K.; Takeuchi S. Beating the Standard Quantum Limit with Four-Entangled Photons. Science 2007, 316, 726–729. 10.1126/science.1138007. 17478715

[ref9] Müller M.; Vural H.; Schneider C.; Rastelli A.; Schmidt O. G.; Höfling S.; Michler P. Quantum-Dot Single-Photon Sources for Entanglement Enhanced Interferometry. Phys. Rev. Lett. 2017, 118, 257402 10.1103/PhysRevLett.118.257402. 28696738

[ref10] Schweickert L.; Jöns K. D.; Zeuner K. D.; Covre da Silva S. F.; Huang H.; Lettner T.; Reindl M.; Zichi J.; Trotta R.; Rastelli A.; Zwiller V. On-Demand Generation of Background-Free Single Photons from a Solid-State Source. Appl. Phys. Lett. 2018, 112, 093106 10.1063/1.5020038.

[ref11] Hanschke L.; Fischer K. A.; Appel S.; Lukin D.; Wierzbowski J.; Sun S.; Trivedi R.; Vučković J.; Finley J. J.; Müller K. Quantum Dot Single-Photon Sources with Ultra-Low Multi-Photon Probability. npj Quantum Information 2018, 4, 43 10.1038/s41534-018-0092-0.

[ref12] Wang H.; et al. On-Demand Semiconductor Source of Entangled Photons Which Simultaneously Has High Fidelity, Efficiency, and Indistinguishability. Phys. Rev. Lett. 2019, 122, 113602 10.1103/PhysRevLett.122.113602. 30951338

[ref13] Liu J.; Su R.; Wei Y.; Yao B.; da Silva S. F. C.; Yu Y.; Iles-Smith J.; Srinivasan K.; Rastelli A.; Li J.; Wang X. A Solid-State Source of Strongly Entangled Photon Pairs with High Brightness and Indistinguishability. Nat. Nanotechnol. 2019, 14, 586–593. 10.1038/s41565-019-0435-9. 31011221 PMC10941235

[ref14] Dousse A.; Suffczyński J.; Beveratos A.; Krebs O.; Lemaître A.; Sagnes I.; Bloch J.; Voisin P.; Senellart P. Ultrabright Source of Entangled Photon Pairs. Nature 2010, 466, 217–220. 10.1038/nature09148. 20613838

[ref15] Müller M.; Bounouar S.; Jöns K. D.; Glässl M.; Michler P. On-Demand Generation of Indistinguishable Polarization-Entangled Photon Pairs. Nat. Photonics 2014, 8, 224 10.1038/nphoton.2013.377.

[ref16] Winik R.; Cogan D.; Don Y.; Schwartz I.; Gantz L.; Schmidgall E. R.; Livneh N.; Rapaport R.; Buks E.; Gershoni D. On-Demand Source of Maximally Entangled Photon Pairs Using the Biexciton-Exciton Radiative Cascade. Phys. Rev. B: Condens. Matter Mater. Phys. 2017, 95, 235435 10.1103/PhysRevB.95.235435.

[ref17] Huber D.; Reindl M.; Covre da Silva S. F.; Schimpf C.; Martín-Sánchez J.; Huang H.; Piredda G.; Edlinger J.; Rastelli A.; Trotta R. Strain-Tunable GaAs Quantum Dot: A Nearly Dephasing-Free Source of Entangled Photon Pairs on Demand. Phys. Rev. Lett. 2018, 121, 033902 10.1103/PhysRevLett.121.033902. 30085806

[ref18] Chen Y.; Zopf M.; Keil R.; Ding F.; Schmidt O. G. Highly-Efficient Extraction of Entangled Photons from Quantum Dots Using a Broadband Optical Antenna. Nat. Commun. 2018, 9, 2994 10.1038/s41467-018-05456-2. 30065263 PMC6068148

[ref19] Zeuner K. D.; Jöns K. D.; Schweickert L.; Reuterskiöld Hedlund C.; Nuñez Lobato C.; Lettner T.; Wang K.; Gyger S.; Schöll E.; Steinhauer S.; Hammar M.; Zwiller V. On-Demand Generation of Entangled Photon Pairs in the Telecom C-Band with InAs Quantum Dots. ACS Photonics 2021, 8, 2337–2344. 10.1021/acsphotonics.1c00504. 34476289 PMC8377713

[ref20] Paul M.; Olbrich F.; Höschele J.; Schreier S.; Kettler J.; Portalupi S. L.; Jetter M.; Michler P. Single-Photon Emission at 1.55 μm from MOVPE-Grown InAs Quantum Dots on InGaAs/GaAs Metamorphic Buffers. Appl. Phys. Lett. 2017, 111, 033102 10.1063/1.4993935.

[ref21] Benson O.; Santori C.; Pelton M.; Yamamoto Y. Regulated and Entangled Photons from a Single Quantum Dot. Phys. Rev. Lett. 2000, 84, 2513–2516. 10.1103/PhysRevLett.84.2513. 11018923

[ref22] da Silva S. F. C.; Undeutsch G.; Lehner B.; Manna S.; Krieger T. M.; Reindl M.; Schimpf C.; Trotta R.; Rastelli A. GaAs Quantum Dots Grown by Droplet Etching Epitaxy as Quantum Light Sources. Appl. Phys. Lett. 2021, 119, 120502 10.1063/5.0057070.

[ref23] Skiba-Szymanska J.; Stevenson R. M.; Varnava C.; Felle M.; Huwer J.; Müller T.; Bennett A. J.; Lee J. P.; Farrer I.; Krysa A. B.; Spencer P.; Goff L. E.; Ritchie D. A.; Heffernan J.; Shields A. J. Universal Growth Scheme for Quantum Dots with Low Fine-Structure Splitting at Various Emission Wavelengths. Phys. Rev. Appl. 2017, 8, 014013 10.1103/PhysRevApplied.8.014013.

[ref24] Zeuner K. Semiconductor Quantum Optics at Telecom Wavelengths. Ph.D. thesis, KTH Royal Institute of Technology, 2020.

[ref25] Trotta R.; Zallo E.; Ortix C.; Atkinson P.; Plumhof J. D.; van den Brink J.; Rastelli A.; Schmidt O. G. Universal Recovery of the Energy-Level Degeneracy of Bright Excitons in InGaAs Quantum Dots without a Structure Symmetry. Phys. Rev. Lett. 2012, 109, 147401 10.1103/PhysRevLett.109.147401. 23083282

[ref26] Plumhof J. D.; Trotta R.; Rastelli A.; Schmidt O. G. Experimental Methods of Post-Growth Tuning of the Excitonic Fine Structure Splitting in Semiconductor Quantum Dots. Nanoscale Res. Lett. 2012, 7, 336 10.1186/1556-276X-7-336. 22726724 PMC3562195

[ref27] Martín-Sánchez J.; et al. Strain-Tuning of the Optical Properties of Semiconductor Nanomaterials by Integration onto Piezoelectric Actuators. Semicond. Sci. Technol. 2018, 33, 013001 10.1088/1361-6641/aa9b53.

[ref28] Trotta R.; Martín-Sánchez J.; Wildmann J. S.; Piredda G.; Reindl M.; Schimpf C.; Zallo E.; Stroj S.; Edlinger J.; Rastelli A. Wavelength-Tunable Sources of Entangled Photons Interfaced with Atomic Vapours. Nat. Commun. 2016, 7, 10375 10.1038/ncomms10375. 26815609 PMC4737804

[ref29] Piredda G.; Stroj S.; Ziss D.; Stangl J.; Trotta R.; Martín-Sánchez J.; Rastelli A. Micro-Machining of PMN-PT Crystals with Ultrashort Laser Pulses. Appl. Phys. A: Mater. Sci. Process. 2019, 125, 201 10.1007/s00339-019-2460-9.

[ref30] Zeuner K. D.; Paul M.; Lettner T.; Reuterskiöld Hedlund C.; Schweickert L.; Steinhauer S.; Yang L.; Zichi J.; Hammar M.; Jöns K. D.; Zwiller V. A Stable Wavelength-Tunable Triggered Source of Single Photons and Cascaded Photon Pairs at the Telecom C-Band. Appl. Phys. Lett. 2018, 112, 173102 10.1063/1.5021483.

[ref31] Bennett A. J.; Pooley M. A.; Stevenson R. M.; Ward M. B.; Patel R. B.; de la Giroday A. B.; Sköld N.; Farrer I.; Nicoll C. A.; Ritchie D. A.; Shields A. J. Electric-Field-Induced Coherent Coupling of the Exciton States in a Single Quantum Dot. Nat. Phys. 2010, 6, 947–950. 10.1038/nphys1780.

[ref32] Lin Z.; Schweickert L.; Gyger S.; Jöns K. D.; Zwiller V. Efficient and Versatile Toolbox for Analysis of Time-Tagged Measurements. J. Instrum. 2021, 16, T08016 10.1088/1748-0221/16/08/T08016.

[ref33] Fokkens T.; Fognini A.; Zwiller V. Optical Quantum Tomography Code; https://github.com/afognini/Tomography/ (accessed 2021-06-06).

[ref34] Huber T.; Predojević A.; Khoshnegar M.; Dalacu D.; Poole P. J.; Majedi H.; Weihs G. Polarization Entangled Photons from Quantum Dots Embedded in Nanowires. Nano Lett. 2014, 14, 7107–7114. 10.1021/nl503581d. 25395237

[ref35] Kolatschek S.; Nawrath C.; Bauer S.; Huang J.; Fischer J.; Sittig R.; Jetter M.; Portalupi S. L.; Michler P. Bright Purcell Enhanced Single-Photon Source in the Telecom O-Band Based on a Quantum Dot in a Circular Bragg Grating. Nano Lett. 2021, 21, 7740–7745. 10.1021/acs.nanolett.1c02647. 34478316

