# Correction to “Strain-Controlled Quantum Dot Fine Structure for Entangled Photon Generation at 1550 nm”

**DOI:** 10.1021/acs.nanolett.2c01688

**Published:** 2022-05-09

**Authors:** Thomas Lettner, Samuel Gyger, Katharina D. Zeuner, Lucas Schweickert, Stephan Steinhauer, Carl Reuterskiöld Hedlund, Sandra Stroj, Armando Rastelli, Mattias Hammar, Rinaldo Trotta, Klaus D. Jöns, Val Zwiller

In the first sentence in section “Acknowledgments”, the grant agreement number of ASCENT+ is incorrect, it should be 871130 instead of 654384.

