# Supplementary material for: Strain-Controlled Quantum Dot Fine Structure for Entangled Photon Generation at 1550 nm
Source: Nano Lett. 2021 Dec 13;21(24):10501–6. doi: 10.1021/acs.nanolett.1c04024 (PMC8704189; doi:10.1021/acs.nanolett.1c04024)
Supplement: Supplementary file 1 — nl1c04024_si_001.pdf [file nl1c04024_si_001.pdf]

Supporting information: Strain-controlled quantum dot fine-structure for entangled-photon generation at 1550 nm

Thomas Lettner,*,† Samuel Gyger,† Katharina D. Zeuner,† Lucas Schweickert,† Stephan Steinhauer,† Carl Reuterskiöld Hedlund,‡ Sandra Stroj,¶ Armando Rastelli,§ Mattias Hammar,‡ Rinaldo Trotta,∥ Klaus D. Jöns,†,⊥ and Val Zwiller*,†

†*Department of Applied Physics, KTH Royal Institute of Technology, Albanova University Centre, Roslagstullsbacken 21, 106 91 Stockholm, Sweden*

‡*Department of Electrical Engineering, KTH Royal Institute of Technology, Electrum 229, 164 40 Kista, Sweden*

¶*Research Center for Microtechnology, Vorarlberg University of Applied Sciences, Campus V, Hochschulstrasse 1, 6850 Dornbirn, Austria*

§*Institute of Semiconductor and Solid State Physics, Johannes Kepler University Linz, 4040, Austria*

∥*Department of Physics, Sapienza University of Rome, Piazzale A. Moro 5, 00185 Rome, Italy*

⊥*Now at: Institute for Photonic Quantum Systems (PhoQS), Center for Optoelectronics and Photonics Paderborn (CeOPP) and Department of Physics, Paderborn University, 33098 Paderborn, Germany*

E-mail: lettner@kth.se; zwiller@kth.se

Contents

List of Figures **S2**

1 Sample growth and device fabrication **S3**

2 Piezoelectric actuator tuning characterization **S4**

3 Second-order correlation measurements **S6**

4 Density matrices for medium and large fine-structure **S8**

List of Figures

S1 Visualization of the tuning behaviour. S5
S2 Exciton auto-correlation measurement result for quantum dot tuned to vanishing FSS. S6
S3 Biexciton auto-correlation measurement and biexciton-exciton cross-correlation measurement to extract the exciton lifetime. S7
S4 Entangled state tomography for medium FSS. S8
S5 Entangled state tomography for high FSS. S8

1 Sample growth and device fabrication

The quantum dot sample is grown by metal-organic vapor-phase epitaxy on GaAs (001) (see Table 1 for a lists of the layers and growth temperatures). We dice the sample into pieces smaller than 2 mm × 2 mm and thin it down to 40 µm thickness by lapping the backside with a mechanical polishing system (Multiprep).

For the following integration onto the piezoelectric actuator we apply SU8 directly to individual actuator legs using a foam head swap. We pick-and-place the thinned sample using an electrostatically-charged plastic tip and hard-bake the SU8 at 220 °C. Then, we use conductive silver paint to glue the piezoelectric actuator to an AlN chip carrier with matching Au contact pads.

Table 1: Layer thicknesses and growth temperatures for the epitaxial sample growth

Layer	Growth temperature [°C]	Thickness [nm]
GaAs	700	100
AlAs	700	200
MMB-layer	700	1100-1520
InAs QDs	530	
Capping $In_{0.3}Ga_{0.7}As$	530	200 - 300

2 Piezoelectric actuator tuning characterization

For the characterization we record polarization resolved µ-PL spectra of the quantum dot while sweeping the voltage for each pair of legs. We then perform peak fitting on the quantum dot emission lines and record the peak position. Next we subtract the trion from the exciton peak position in order to correct for temperature-related spectral shift introduced by the cryostat. From a fit of the data to a sine function we extract the fine-structure splitting (FSS) from the fit value of the amplitude (FSS is twice the amplitude).

The phase is related to the strain anisotropy angle ϕ of the in-plane strain tensor. In accordance to the definition of the anisotropy vector, we can then visualize the fine-structure splitting in Cartesian coordinates (see Fig. S1) using:

$$\mathrm{FSS}_y = \mathrm{FSS} * \sin(2\phi) \tag{1}$$

$$\mathrm{FSS}_x = \mathrm{FSS} * \cos(2\phi) \tag{2}$$

We observe that the uniaxial strain induced by the six-legged actuator results in a change of this vector along a straight line in this representation. The line characterizes the tuning direction and range for a specific leg of the actuator.

Minimizing the magnitude of FSS requires aligning the strain aniostropy angle with the actuation direction using a second leg. Knowledge of the straining directions of the legs allows to predict how each leg presets the anisotropy angle. One leg can then set the angle in order to match the tuning direction of a second leg with sufficient tuning range to reach zero FSS.

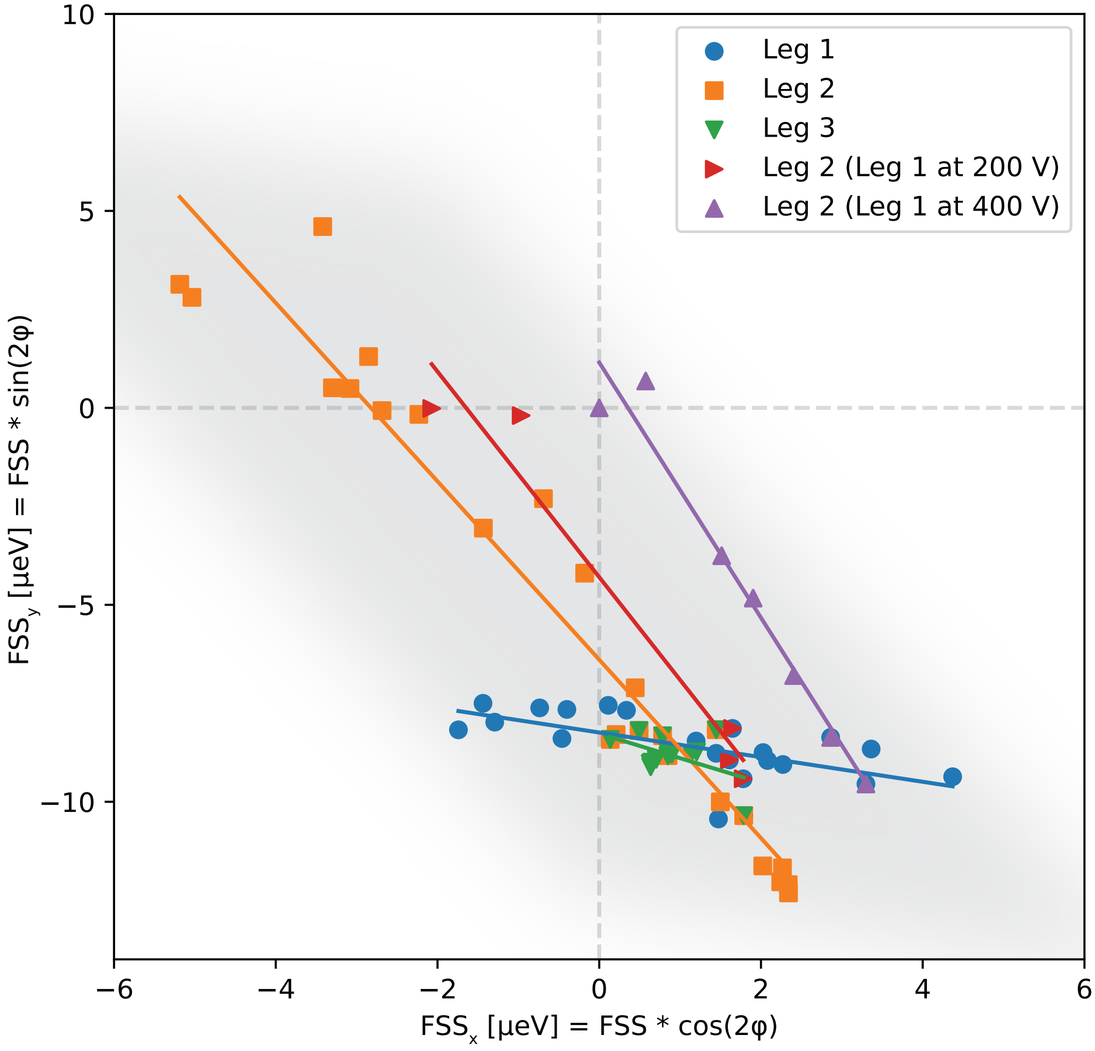

Figure S1: Visualization of the tuning behaviour. Points represent the FSS projection, with magnitude and angle extracted from polarization-resolved µ-PL spectra. Lines are linear fits indicating the effect of the uniaxial strain induced by the actuator. Combinations of legs (red and violet) allow to access regions of low FSS. The shaded area indicates approximately the tunable region for the FSS when using leg 1 and leg 2 together.

3 Second-order correlation measurements

We measure exciton auto-correlation using a fiber beam-splitter with the outputs connected to two single-photon detectors. The input of the beam-splitter connects to the exciton output from the state tomography measurement setup, which comprises transmission grating, waveplates and polarizer for polarization control. We characterize our setup efficiency to 5.7% and derive from this 0.6% collection efficiency into the first lens.

Next we tune the quantum dot to vanishing FSS and obtain the exciton auto-correlation histogram with the characteristic absence of the central peak at time-delay zero (Fig. S2)). We calculate $g^{(2)}(0) = 0.009 \pm 0.001$ for the second-order correlation function at time-delay zero, by integrating the coincidence counts in a 6 ns time-window around the peak centers and then dividing the result for the central peak by the average integrated counts of the side peaks.

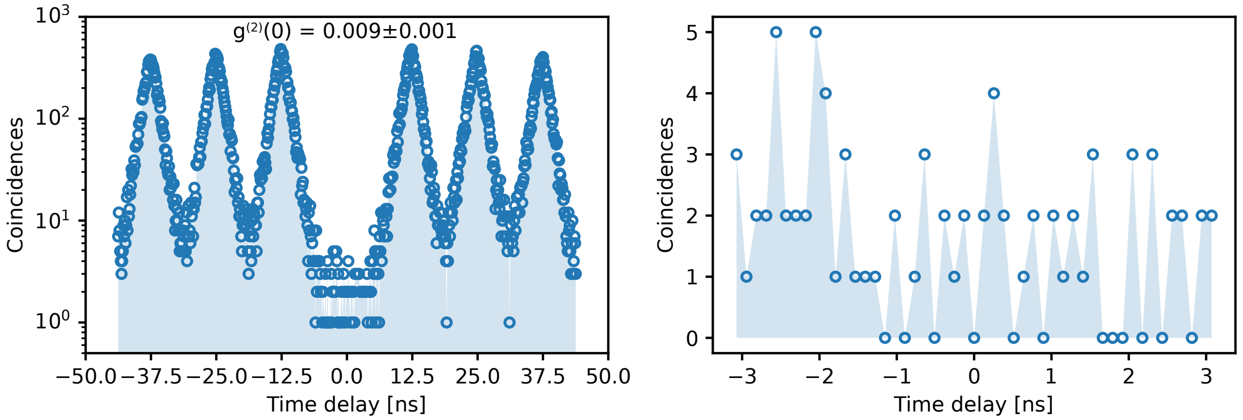

Figure S2: Exciton auto-correlation measurement result for quantum dot tuned to vanishing FSS. Left: Central and side peaks (logarithmic scale). Right: Zoom-in on the central peak (linear scale).

In comparison, for the biexciton auto-correlation histogram shown in the left panel of Figure S3 we obtain the value $g^{(2)}(0)_{XX} = 0.040 \pm 0.004$. Finally, we extract also the exciton lifetime from biexciton-exciton cross-correlation measurements performed in the HH and HV basis, and obtain $\tau_X = (963 \pm 40)$ ps by fitting the combined coincidences to an exponential decay e^{-t/τ_X} (right panel in Fig. S3).

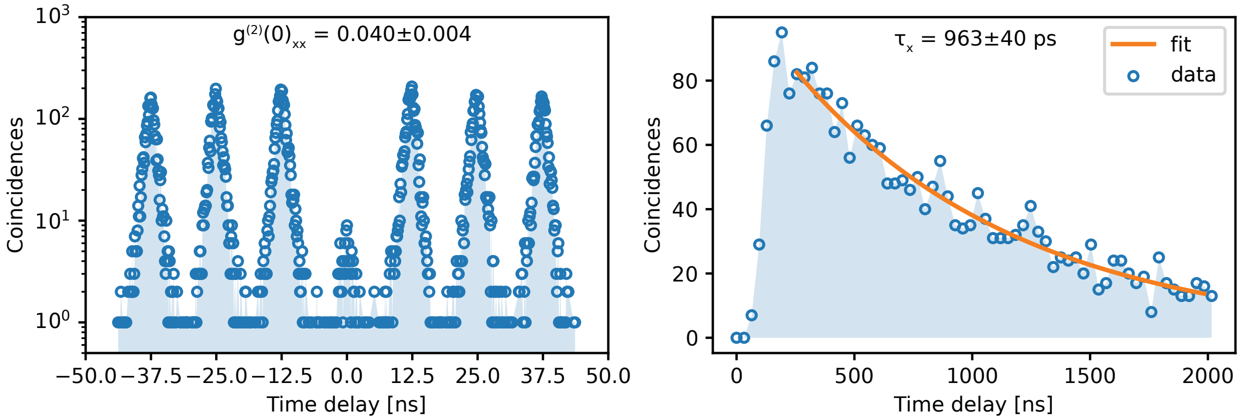

Figure S3: Biexciton auto-correlation measurement and biexciton-exciton cross-correlation measurement to extract the exciton lifetime. Left: Biexciton auto-correlation histogram with central and side peaks (logarithmic scale). Right: Cross-correlation histogram data and fit to an exponential decay.

4 Density matrices for medium and large fine-structure

In addition to the low FSS case, we can also visualize the density matrix for time bin 96 ps of the time-resolved two-photon state tomography for the time bin at 96 ps with 32 ps binning width (Fig. S4 and Fig. S5) and achieve similar peak fidelities of 83% and 85%, for medium and high FSS.

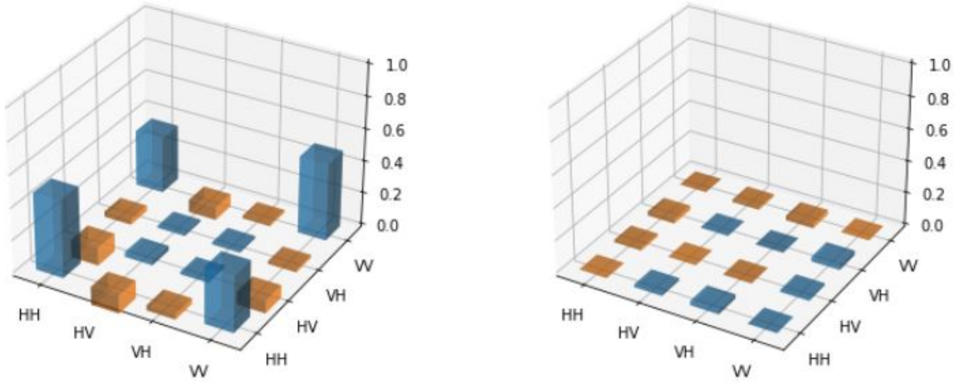

Figure S4: Entangled state tomography for medium FSS. Real (left) and imaginary (right) parts of the density matrix with peak fidelity of 83% for the medium FSS setting corresponding to 6.5 µeV.

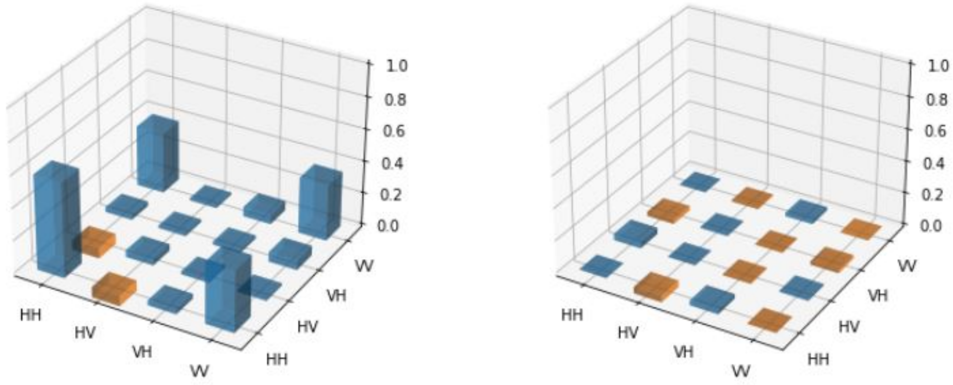

Figure S5: Entangled state tomography for high FSS. Real (left) and imaginary (right) parts of the density matrix with peak fidelity of 85% for the high FSS setting corresponding to 12.9 µeV

.
